# Hemoglobin stimulates vigorous growth of *Streptococcus pneumoniae* and shapes the pathogen's global transcriptome

**DOI:** 10.1038/s41598-020-71910-1

**Published:** 2020-09-16

**Authors:** Fahmina Akhter, Edroyal Womack, Jorge E. Vidal, Yoann Le Breton, Kevin S. McIver, Shrikant Pawar, Zehava Eichenbaum

**Affiliations:** 1 Department of Biology, Georgia State University, Atlanta, GA USA; 2 Department of Microbiology and Immunology, University of Mississippi Medical Center, Jackson, MS USA; 3 Department of Cell Biology and Molecular Genetics, Maryland Pathogen Research Institute, University of Maryland, College Park (UMCP), College Park, MD USA; 4 Present Address: Wound Infections Department, Bacterial Diseases Branch, The Walter Reed Army Institute of Research, Silver Spring, MD USA; 5 Present Address: Yale Center for Genome Analysis, Yale University, New Haven, CT USA

**Keywords:** Bacterial genetics, Pathogens

## Abstract

*Streptococcus pneumoniae* (Spn) must acquire iron from the host to establish infection. We examined the impact of hemoglobin, the largest iron reservoir in the body, on pneumococcal physiology. Supplementation with hemoglobin allowed Spn to resume growth in an iron-deplete medium. Pneumococcal growth with hemoglobin was unusually robust, exhibiting a prolonged logarithmic growth, higher biomass, and extended viability in both iron-deplete and standard medium. We observed the hemoglobin-dependent response in multiple serotypes, but not with other host proteins, free iron, or heme. Remarkably, hemoglobin induced a sizable transcriptome remodeling, effecting virulence and metabolism in particular genes facilitating host glycoconjugates use. Accordingly, Spn was more adapted to grow on the human α − 1 acid glycoprotein as a sugar source with hemoglobin. A mutant in the hemoglobin/heme-binding protein Spbhp-37 was impaired for growth on heme and hemoglobin iron. The mutant exhibited reduced growth and iron content when grown in THYB and hemoglobin. In summary, the data show that hemoglobin is highly beneficial for Spn cultivation in vitro and suggest that hemoglobin might drive the pathogen adaptation in vivo. The hemoglobin receptor, Spbhp-37, plays a role in mediating the positive influence of hemoglobin. These novel findings provide intriguing insights into pneumococcal interactions with its obligate human host.

## Introduction

*Streptococcus pneumoniae* (Spn) is a significant human pathogen that causes illnesses ranging in severity from common otitis media infections to invasive diseases such as pneumonia, bacteremia, and meningitis. Pneumococcal pneumonia is also a significant risk factor for the development of cardiac diseases and heart failure^1,2^. Altogether, the toll of Spn on human health is substantial, and the pathogen is responsible for ~ 15 million infections each year and about half a million deaths in children worldwide^3,4^. Pneumococci commonly colonize the human nasopharynx, and Spn can persist asymptomatically in healthy individuals for several weeks and up to a few months^5^. From the nasopharynx, Spn can be transmitted among hosts^6^ and spread to other organs. Young children (< 5 years of age), the elderly, and immunocompromised persons are the most susceptible individuals to pneumococcal infections^7–9^. While colonization of the upper respiratory tract is a pre-requisite for pneumococcal pathogenesis and infectivity, the factors that govern the establishment of Spn in the human host and the pathogen's transition into a virulent state, are not fully appreciated.

Healthy individuals typically avoid infection by *S. pneumoniae*. Still, the opportunistic pathogen can thrive in susceptible hosts and cause serious ailments. *S. pneumoniae,* though, is a fastidious bacterium that in vitro exhibits relatively weak growth. Several studies were undertaken to optimize streptococcal cultivation in a complex (e.g., Todd-Hewitt broth containing yeast extract (THYB) and Tryptic Soy Broth) or defined media^10–12^. Pre-cultivation (i.e., inoculating with cells collected from a logarithmic-phase culture), medium replenishment, and growth in bioreactors with controlled growth parameters (such as pH and oxygen levels) were found to improve pneumococcal growth, although often in a strain-dependent manner^10^. Inoculating THYB with cells grown overnight in either broth or solid medium typically results in a long lag period that is followed by a relatively short exponential growth. The culture enters the stationary phase with low optical density (O.D._600_ of 0.3–0.5, see references^13,14^ for examples). In addition to the limited biomass, Spn cultures remain viable for a much shorter time when compared to other bacteria. An autolysis mechanism that is activated upon entry into the stationary phase or in response to inhibition of the cell-wall synthesis is often responsible for the decreased viability observed during in vitro cultivation. A threshold concentration of the extracellular amidase, LytA, determines the onset of autolysis. During infection, however, the cell-wall degrading LytA is needed for fratricidal lysis and virulence^15,16^. The pneumococcal pyruvate oxidase, SpxB, and its metabolic by-product H_2_O_2_ also contribute to pneumococcal killing during the stationary phase of growth^17^.

Within the human body, high-affinity proteins sequester iron and reduce the metal bioavailability for invading microbes in a process called nutritional immunity. During colonization and the courses of infections, pneumococci must gain access and retrieve the iron it needs for growth from host proteins that carry heme or metal iron^18–20^. Most of the metal in the human body is in heme within the erythrocyte hemoglobin (67% of the total body iron). Myoglobin and cytochrome represent most of the remaining heme pool (3.5 and 3%). Iron is also highly sequestered in the extracellular compartment. The serum proteins hemopexin and transferrin respectively remove the small amounts of free heme and iron in the serum. The host lactoferrin sequesters ferric ions from secretions and near the infection site. Spn readily grows on heme or hemoglobin iron^21^ but is unable to obtain the metal from transferrin and lactoferrin^21^. The pneumococcal mechanisms that facilitate the capture and uptake of heme are only partially understood^22,23^. The ABC transporter PiuBCDA (also known as Pit1BCDA) is the only recognized heme importer in Spn. PiuA, the ligand-binding component, binds heme and hemoglobin in vitro*,* and inactivation of the *piuBCDA* genes partially impairs Spn growth on heme iron^24^. Spbhp-37, is the second substrate-binding protein in Spn that interacts with hemoglobin and heme^25^. Spbhp-37 antiserum inhibits pneumococcal growth on hemoglobin iron. However, a mutant in *spbhp-37* was not described, and it's not known which transporter works with this substrate-binding protein for heme import. A recent report implicated an additional Spn protein, Spbhp-22, in iron uptake. In vitro assays showed that Spbhp-22 binds heme*,* but the mechanism by which this cytoplasmic protein promotes iron or heme intake remains unclear^26^. Since hemoglobin is the primary source of iron during infection, we hypothesized that hemoglobin is vital for pneumococcal pathophysiology.

## Results

### Hemoglobin stimulates unusually robust growth of Spn in batch cultures

**Figure 1:**
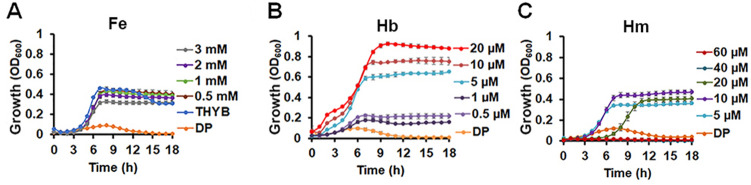
Hemoglobin-dependent growth of Spn D39 in iron-deplete medium. THYB was inoculated with D39 cells grown on BAPs (18 h, starting O.D._600_ = 0.05). Shown is growth in (**A**) THYB, THYB with 3 mM 2, 2′-Dipyridyl (DP), THYB with DP and 0.5–3 mM of FeNO_3_ (Fe); (**B**) THYB with DP supplemented with 0.5 µM -20 µM hemoglobin (Hb); and (**C**) THYB with DP supplemented with 5–60 µM heme (Hm). The data are representative of three independent experiments performed in triplicates; error bars indicate SD.

To test the use of hemoglobin iron by Spn, we adopted the growth assay from our studies with the related Group A Streptococcus^27^. We cultivated Spn in fresh THYB, with or without the iron chelator 2, 2′-Dipyridyl (DP), and different iron supplements (hemoglobin, heme, and FeNO_3_) (Fig. 1). Spn did not grow in THYB-DP, but growth was restored when we supplemented the medium with ferric iron (≥ 0.5 mM, Fig. 1A), demonstrating that DP prevents pneumococcal cultivation by limiting iron bioavailability. Supplementation with hemoglobin (Fig. 1B) or heme (Fig. 1C) also supported Spn growth in THYB-DP in a dose-dependent manner. Higher amounts of FeNO_3_ compared to a heme source (mM vs. μM, respectively) were needed to support growth, because DP chelates metal iron but not heme^27,28^. Surprisingly, hemoglobin facilitated a much better growth in THYB-DP compared with free iron (Fig. 1B). In the presence of hemoglobin, the culture displayed only a brief lag phase, which was followed by a prolonged (and somewhat bi-phasic) exponential period, reaching a higher maximal optical density. Free heme also supports Spn growth in THYB-DP, but growth was not as robust as with hemoglobin, and heme became a growth-inhibitory above ten µM (Fig. 1C).

**Figure 2:**
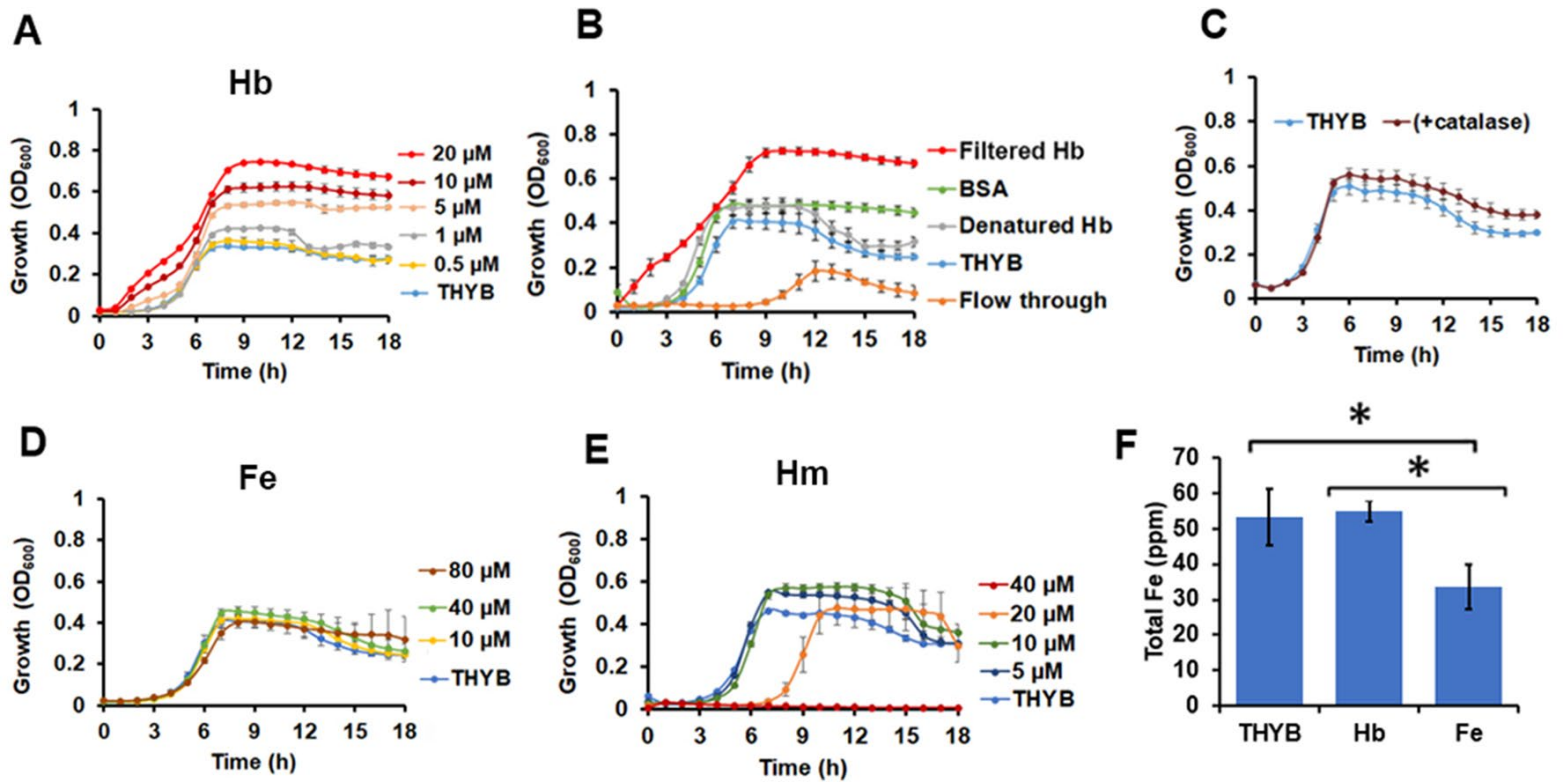
Hemoglobin stimulates a robust growth of Spn D39 in standard (iron-complete) medium. THYB was inoculated with D39 cells grown on BAPs (18 h, starting O.D._600_ = 0.05. Shown is growth in (**A**) THYB with 0–20 µM hemoglobin (Hb); (**B**) THYB with 20 µM BSA, denatured hemoglobin, filtered hemoglobin, or the flow-through; (**C**) THYB with or without catalase. (**D**) THYB supplemented with 0–80 µM FeNO_3_ (Fe); (**E**) THYB with 0–40 µM heme (Hm). The data are representative of three independent experiments performed in triplicates; error bars indicate SD. (**F**) Total intracellular iron content measured by ICP-MS in culture samples (normalized to optical density) grown in THYB, THYB with 20 µM hemoglobin (Hb), or THYB with 80 µM FeNO_3_ (Fe). The data represents the average of three independent biological replicates; error bars indicate SD. The asterisks denote statistical significance, *P* ≤ 0.05 (THYB vs. Fe, and Hb vs. Fe, Student's t-test).

Spn growth in THYB-DP with hemoglobin (≥ 5 µM) exceeded the one observed in standard medium (THYB, Fig. 1A,B). We tested the impact of adding 0.5–20 µM hemoglobin to iron complete THYB. Remarkably, hemoglobin stirred vigorous growth in a dose-dependent manner (Fig. 2A). Incubation with 20 µM serum albumin, which has a similar molecular weight (~ 60 kDa), resulted only in a minimal growth improvement (Fig. 2B). Supplementation with hemoglobin that was heat-inactivated did not promote growth. To rule out the possibility that a contaminant in our hemoglobin preparation was responsible for the observed enhanced growth, we filtered the hemoglobin solution using 10,000 MW cutoff and tested both fractions for growth impact. The filtered hemoglobin retained activity while the flow-through was somewhat growth-inhibitory (Fig. 2B). It seemed possible that the positive growth induced by hemoglobin results from the intrinsic peroxidase activity of hemoglobin^29^, leading to protection from hydrogen peroxide^30^. To test this hypothesis, we grew Spn in THYB and added catalase to scavenge hydrogen peroxide. Results in Fig. 2C demonstrates that supplementation with catalase had a minimal impact on Spn cultivation.

Supplementation with an equimolar amount of iron (10–80 µM FeNO_3_, each hemoglobin molecule has four heme groups) did not have a significant effect (Fig. 2D). The addition of free heme to THYB did not improve growth at the low µM range and was inhibitory above ten µM (Fig. 2E). To evaluate whether the enhanced growth was due to an improved uptake of iron when pneumococci are incubated with hemoglobin, we measured intracellular iron levels by ICP-MS (Fig. 2F). Equal amounts of iron were found in cells grown in THYB or THYB with 20 μM hemoglobin. Surprisingly, supplementation with 80 μM FeNO_3_ resulted in an actual reduction in the total iron cellular level.

We next tested the viability of cells at different time points during growth in THYB with and without 20 µM hemoglobin. Consistent with the higher optical density, in the presence of hemoglobin, viable count at 8-h post-inoculation was about twice that of cultures grown without hemoglobin (Table 1). Cells exhibited a sharp decline in viability after overnight incubation (18 h) in both THYB and THYB with hemoglobin. However, 18-fold more viable cells were obtained in cultures grown with hemoglobin compared to cultures cultivated in THYB alone (Table 1, 18 h). In summary, hemoglobin-induced growth and viability are a hemoglobin-dependent phenomenon observed in both iron-deplete and iron-complete medium.

**Table 1:** Effect of hemoglobin supplementation on Spn growth.

Incubation time (h)	Conditions^a^	Total (CFU/ml)
8	NS	2.6 ± 1.3 × 10^8^
	Hb	5.5 ± 1.1 × 10^8^
18	NS	4.7 ± 3.1 × 10^2^
	Hb	8.5 ± 6.4 × 10^3^

^a^NS denotes THYB without supplements, Hb denotes THYB supplemented with 20 μM hemoglobin. Statistical analysis: 8 h, *P* = *0.02*; 18 h, *P* = *0.04* (Student’s t-test).

### Hemoglobin modulates Spn growth independently of the strain or the growth assay

**Figure 3:**
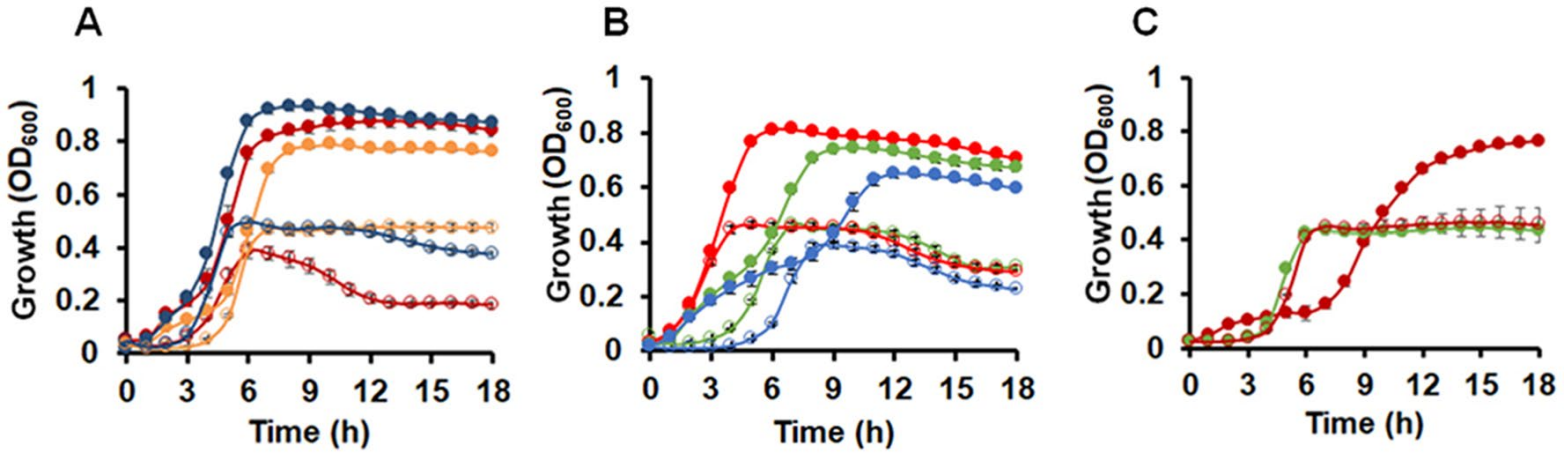
Hemoglobin stimulates Spn growth independently of the strain or the growth assay. Shown is Spn growth in fresh THYB (empty symbols) or THYB with 20 µM hemoglobin (full symbols). The culture starting O.D._600_ is indicated. (**A**) TIGR4 (red), and the clinical isolates 3,875 (blue), and 8,655 (yellow) grown on BAPs (18 h) was used as the inoculum (O.D._600_ = 0.05). (**B**) THYB was inoculated with D39 cells from frozen logarithmic cultures (O.D._600_ = 0.02, red), THYB cultures (18 h, O.D._600_ = 0.05, blue), or cell from BAPs (18 h, O.D._600_ = 0.05, green). (**C**) D39 growth in CAT medium (empty symbols), CAT medium with 20 µM hemoglobin (red, full symbols) or 20 µM human serum albumin (green, full symbols). The data are representative of three independent experiments performed in triplicates; error bars indicate SD.

Next, we asked if the response to hemoglobin is widespread among pneumococcal strains, and thereby tested the impact of hemoglobin on TIGR4 and two clinical isolates. These experiments revealed that hemoglobin stimulated growth in all three strains (Fig. 3A). The positive impact of hemoglobin might only be seen in our experimental system whereby Spn are grown on blood agar plates prior to inoculating THYB. To examine if this is the case, we inoculated fresh THYB with an overnight culture grown in THYB (Fig. 3B). Compared to cells collected from BAPs, bacteria that came from liquid culture (in THYB) exhibited a more prolonged lag phase and yielded lower final biomass. Nonetheless, the addition of hemoglobin still significantly improved the second round of growth in THYB in these cells. We next used frozen logarithmic cells grown in THYB to start new THYB cultures with and without hemoglobin (Fig. 3B). Inoculating the medium with logarithmic cells abolished the lag phase and improved overall growth. Still, supplementation with hemoglobin enhanced growth even further compared to THYB alone. We also tested the effect of hemoglobin on pneumococcal growth in Casein-Tryptone (CAT) medium. When we inoculated CAT medium with Spn cells collected from BAPs, the bacterial growth was comparable to the one seen in THYB inoculated with cells from BAPs. The addition of hemoglobin to CAT, however, altered the bacterial growth pattern and yield. In the presence of hemoglobin, the pneumococci exhibited only limited growth for the first six hours in CAT. This initial phase was followed by extended exponential growth that resulted in higher biomass compared to pneumococci growing in CAT only (Fig. 3C). Supplementing with human serum albumin in CAT as a control exhibited similar growth as seen in THYB (Fig. 3C). In summary, hemoglobin dramatically impacts growth in batch cultures of multiple pneumococcal strains regardless of the assay, or media used.

### Hemoglobin induced a sizable transcriptome remodeling

**Figure 4:**
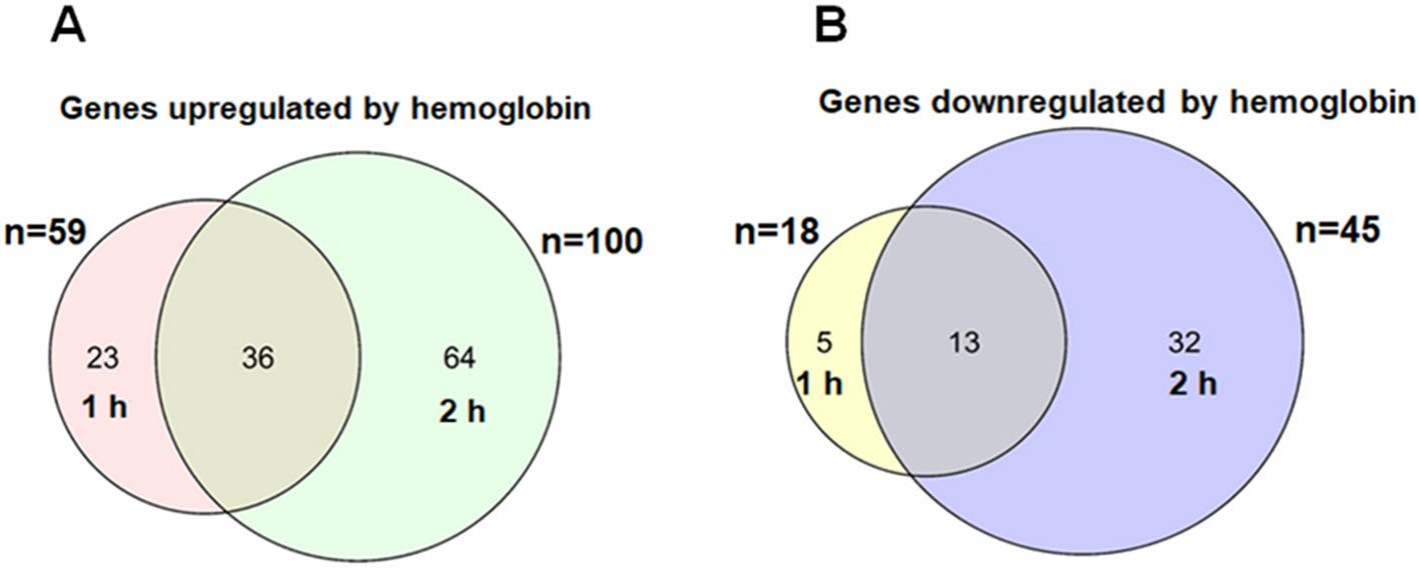
The addition of hemoglobin to the culture medium triggers a significant transcriptome remodeling in Spn. Venn diagram (using R) of differentially expressed genes in D39 culture (fold change ≥ 2). (**A**) Genes up-regulated by hemoglobin. (**B**) Genes down-regulated by hemoglobin.

Since hemoglobin has a considerable influence on growth, we asked how it may affect pneumococcal gene expression. Hemoglobin or saline (as a control) was added to Spn cultures grown in THYB at the early log phase and culture samples were collected one- and two-hours post-treatment. RNA was isolated from four biological replicates for each growth condition and analyzed by RNA-Seq. This global transcriptome study revealed that the addition of hemoglobin to the growth medium resulted in a significant shift in gene expression (Supplementary Table S1). 59 Spn genes were up-regulated and 18 genes were down-regulated (at least two-fold) in response to hemoglobin within one hour (Fig. 4A,B), and a total of 100 and 45 genes were induced or repressed respectively two hours post-treatment. qRT-PCR on a subset of regulated genes validated the RNA seq findings (Fig. S1).

**Figure 5:**
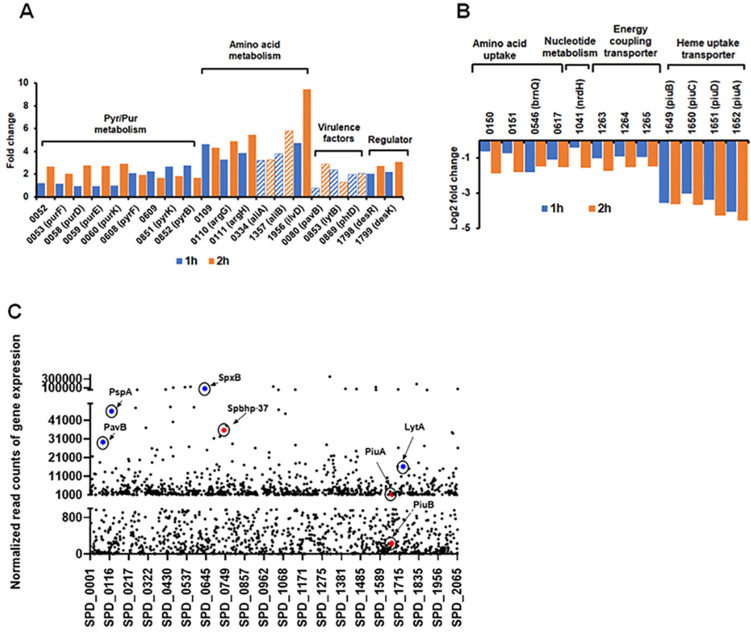
Hemoglobin activates Spn genes vital for host colonization. The relative expression of genes involved in metabolism, nutrient uptake, virulence, or regulation, at 1 h and 2 h post hemoglobin addition (Y-axis) is plotted for D39 genes (X-axis). (**A**) Up-regulated genes. Stripes indicate involvement in nasopharyngeal colonization. (**B**) Down-regulated genes. (**C**) Dot plot representation of gene expression levels depicting average normalized RNA-seq read counts (Y-axis) for cells with 1 h post-hemoglobin treatment is plotted for D39 genes (X-axis). Genes encoding heme/hemoglobin binding (red) or virulence factors (blue) are highlighted.

Many of the genes that were differentially expressed in response to hemoglobin are related to the import and biosynthesis of nucleotides, amino acids, fatty acids, and lipids (Fig. 5). Notably, hemoglobin activated the transcription of several metabolic genes and genes encoding virulence factors needed for Spn nasopharyngeal colonization and infections. These include the genes of the oligopeptide-binding proteins, *aliA* and *aliB*^31^, the PGN hydrolase, *lytB*^32^, the gene encoding the pneumococcal histidine triad protein D, *phtD*^33^ and the surface adhesin, *pavB* (Fig. 5A)^34^. Hemoglobin also repressed many metabolic genes of which, it is noteworthy that the whole operon coding for proteins of a heme importer, *piuBCDA,* was down-regulated by incubation with hemoglobin (SPD_1649-52, Fig. 5B)^35^. The expression of the heme/hemoglobin binding protein, *spbhp-37,* which also promotes Spn use of hemoglobin iron^25,36^, was high but not differentially expressed (Fig. 5C).

**Figure 6:**
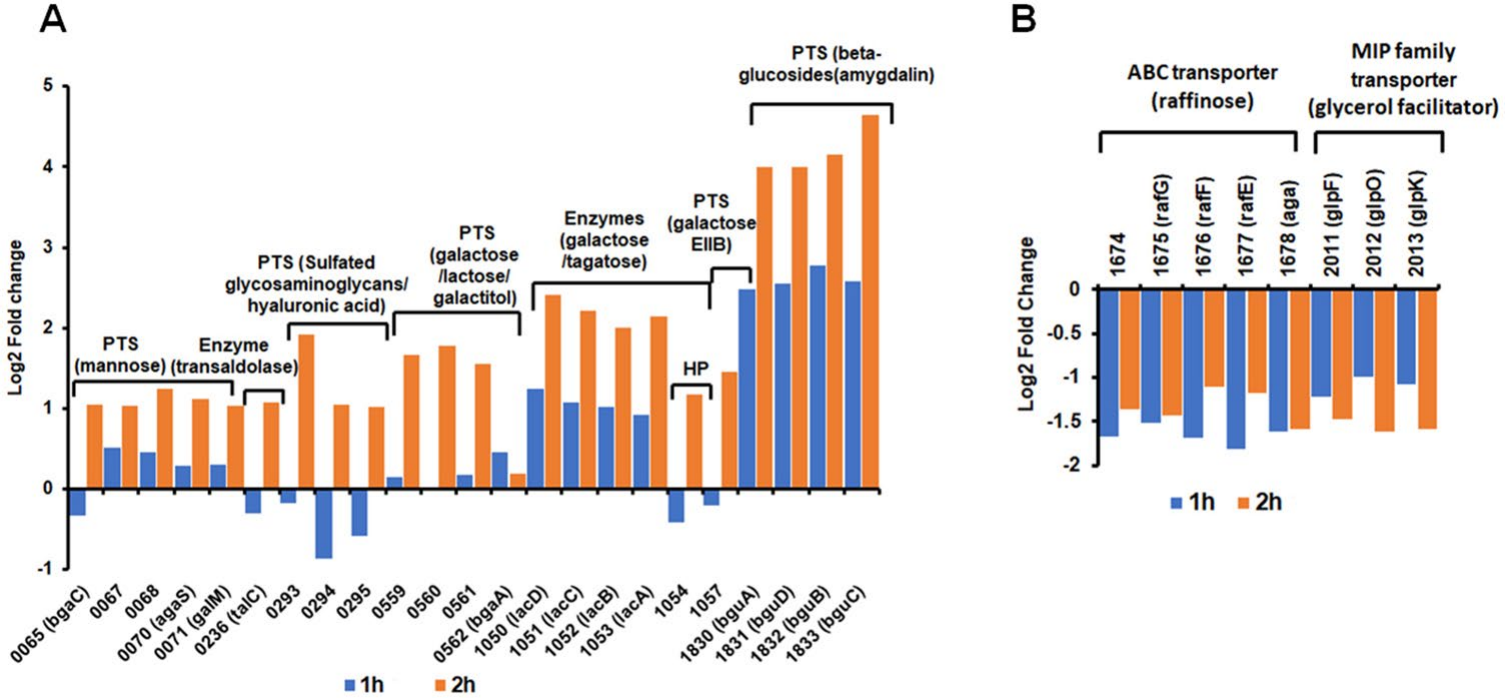
Hemoglobin up-regulates Spn genes involved in host glycoconjugate use. Log_2_-fold changes in gene expression levels (Y-axis) is plotted for D39 genes (X-axis). (**A**) PTSs, enzymes, and hypothetical protein (HP). (**B**) down-regulated sugar transporters.

Many of the hemoglobin-responding genes are related to carbohydrate intake and conversion (Fig. 6). The *bgu* operon^37^ encoding a lactose type phosphotransferase system (PTS) (SPD_1830-33) exhibited the most robust induction post hemoglobin addition (sixfold and 16–25-fold in the first and second hours respectively, Fig. 6A). In vitro, this PTS system mediates the use of beta-glucosides such as amygdalin or cellobiose^38^. During infection, however, β-linked disaccharides found in glycosaminoglycans of the host extracellular matrix are the proposed substrates of the *bgu* PTS. Other hemoglobin responding loci involved in carbohydrate metabolism include the *lacABCD* genes (SPD_1050-54, the tagatose pathway enzymes), *bgaA* and *bgaC* enzymes, and the associated galactose and mannose type PTS systems (SPD_0559-62 and 0,067–71 respectively). A mannose-type PTS system (SPD_0293-5) that is involved in the use of sulfated-glycosaminoglycan (e.g., hyaluronic acid)^38^ was up-regulated in the second hour of hemoglobin treatment (Fig. 6A). In contrast, hemoglobin down-regulated the expression of the ABC transporter (*rafGFE*) that imports the trisaccharide raffinose and of a glycerol facilitator (Fig. 6B).

### Hemoglobin promotes pneumococcal growth on host glycoconjugates in vitro

**Figure 7:**
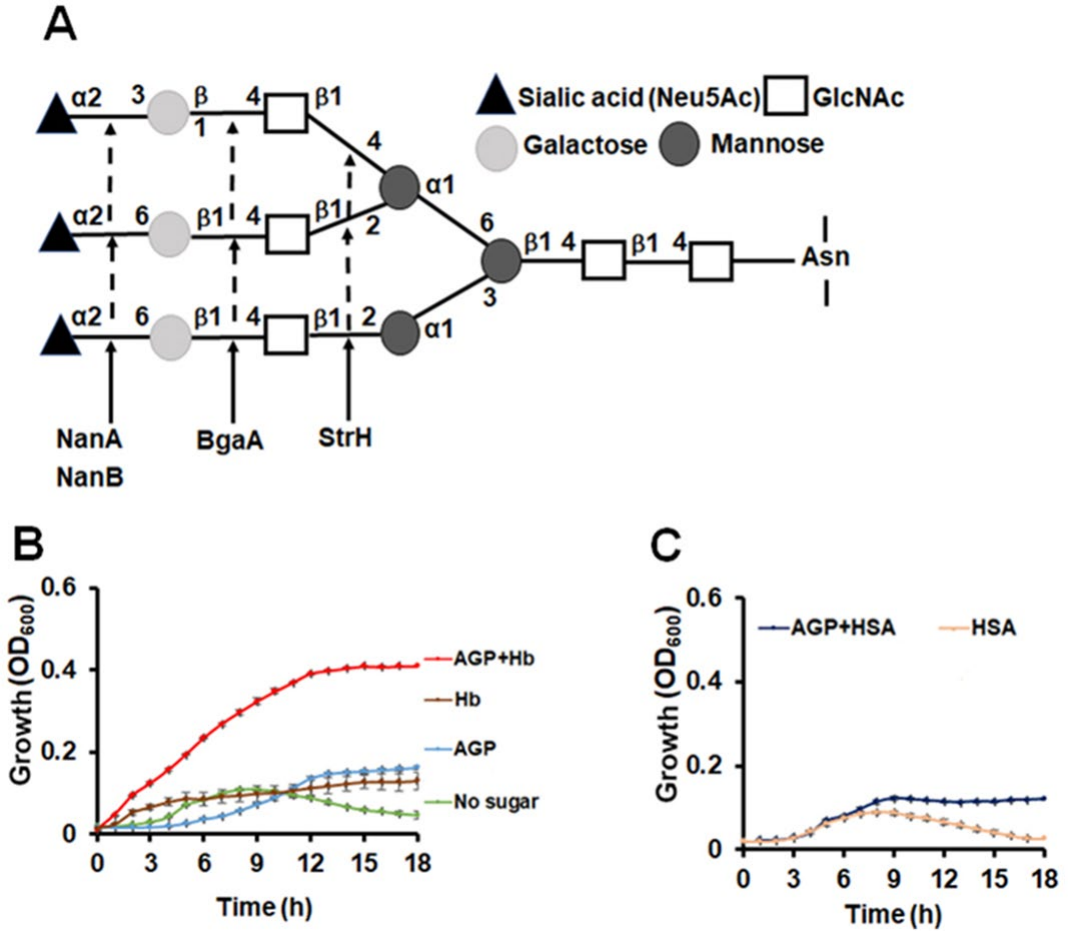
Hemoglobin facilitates Spn growth on a human glycoprotein. (**A**) Schematic representation of the human α − 1-acid glycoprotein (AGP), as described in^58^. Arrows indicate the cleavage sites of the Spn enzymes, neuraminidase (NanA), galactosidase (BgaA), and N-acetylglucosaminidase (StrH). Fresh medium was inoculated with D39 grown on BAPs (18 h, starting O.D._600_ = 0.05). (**B**) Shown is Spn growth in sugar-free CAT medium (no glucose added), or sugar-free CAT with 5 mg/ml AGP, and/or 20 µM hemoglobin (+ Hb). (**C**) The same as in (A), only that 20 µM human serum albumin (+ HSA) was added instead of hemoglobin. The data are representative of three independent experiments performed in triplicates; error bars indicate SD.

Since hemoglobin induced the expression of PTSs and enzymes that are known or predicted to be involved in the use of host glycans, we hypothesized that hemoglobin enhances the ability of Spn to use these host molecules as nutrients. To test this hypothesis, we examined the impact of hemoglobin on Spn growth on the human α − 1-acid glycoprotein, AGP (N-linked glycan, Fig. 7A). Spn growth in CAT medium (which contains glucose) was similar to that seen in THYB (Fig. 3C). The removal of the glucose from the CAT broth, however, impeded growth (Fig. 7B). Spn grew rapidly when the sugar-free CAT was supplemented with both AGP and hemoglobin, but not in sugar-free CAT medium supplemented with either AGP or hemoglobin individually (Fig. 7B). The addition of human serum albumin as a control did not promote growth in sugar-free CAT with or without AGP (Fig. 7C). These data indicate that hemoglobin promotes pneumococcal use of AGP in vitro and suggests it may enhance the use of host glycoproteins as a carbohydrate source in vivo.

### A *spbhp-37* deletion mutant exhibits a lessened growth response to hemoglobin

**Figure 8:**
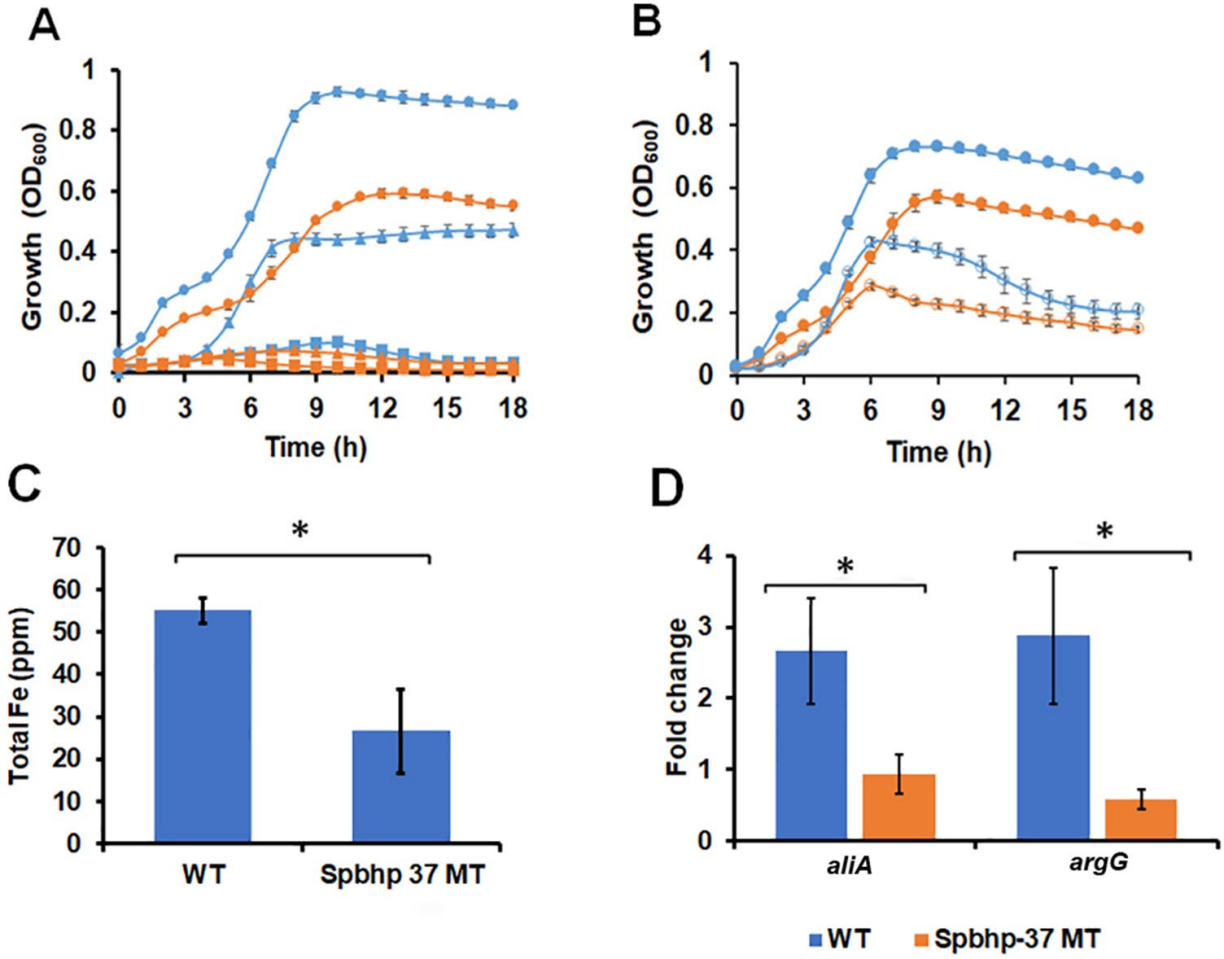
The heme/hemoglobin binding protein, Spbhp-37, plays a role in mediating the positive impact of hemoglobin in Spn. Fresh medium was inoculated with D39 (blue) and the isogenic Δ*spbhp-37* strain (orange) grown on BAPs (18 h, starting O.D.600 = 0.05). Shown is growth in (**A**) THYB with 3 mM 2, 2′-Dipyridyl (DP), THYB with DP and 10 μM heme (triangles)  or 20 μM hemoglobin (circles), or (**B**) THYB (empty symbols) or THYB with 20 µM hemoglobin (full symbol). The data are representative of three independent experiments performed in triplicates; error bars indicate SD. (**C**) Total intracellular iron content (ppm) determined by ICP-MS in D39 wild type and Spbhp-37 mutant cultures samples (normalized to optical density) grown in THYB with 20 µM hemoglobin. The data represents the average of three independent biological replicates; error bars indicate SD. (**D**) Fold change in gene expression in the wild type and Δ*spbhp-37* strains 2 h post hemoglobin treatment relative to the control (saline) as determined by qRT-PCR. The experiments were performed in duplicates with at least two biological replicates. The replicates data are shown as the mean ± SD. The asterisks denote statistical significance, *P* ≤ 0.05 (WT vs. MT, Student's t-test).

Although hemoglobin stimulates Spn growth in both iron-complete and iron-deplete THYB, it still seemed possible that the positive influence of hemoglobin is related to its ability to donate heme. THYB supplementation with hemoglobin repressed transcription of the heme importer genes *piuBCDA,* allowing for only very low expression (Fig. 5C). The expression of *spbhp-37,* the second heme/hemoglobin receptor with a role in heme uptake, did not change in response to hemoglobin*.* Still, *spbhp-37* transcription was relatively high under our experimental condition (Fig. 5C). In silico analysis predicted that Spbhp-37 is expressed as a monocistronic mRNA^39^. To see if Spbhp-37 plays a role in the response, we generated a deletion mutant (replacing *spbhp-37* ORF with that of *ermB)* and tested its impact on hemoglobin growth induction (Fig. 8). The Δ*spbhp-37* mutant was not able to grow in THYB-DP supplemented with free heme and exhibited growth attenuation when grown in THYB-DP supplemented with hemoglobin (Fig. 8A). The mutant also had a growth phenotype in THYB alone. Although Δ*spbhp-37* growth was improved in the presence of hemoglobin, it was still reduced compared with the wild type strain (Fig. 8B). ICP-MS analysis demonstrated a significant reduction in total iron levels in the Δ*spbhp-37* compare with the parental strain in cells grown in THYB with 20 μM hemoglobin (Fig. 8C). qRT-PCR analysis demonstrated that the expression of the *aliA* and *argG* genes was not induced in response to hemoglobin in the Δ*spbhp-37* mutant as it did in the wildtype strain (Fig. 8D). These observations confirm a role for Spbhp-37 in iron uptake and suggest that this hemoglobin/heme-binding protein plays a role in the Spn response to hemoglobin.

## Discussion

In this study, we demonstrated that hemoglobin, a host hemoprotein, has a substantial impact on Spn physiology and planktonic growth. The data reveal that the presence of hemoglobin greatly benefits pneumococcal growth and viability in complex laboratory media, redirecting the pneumococcal transcriptome and metabolic capacity. This appears to enhance the ability of Spn to use host glycoproteins as a source of carbohydrates, providing a potential fitness advantage during colonization and infection. The data also suggest that the heme uptake protein, Spbhp-37, plays a role in the positive influence of hemoglobin on Spn physiology. Below we discuss how these intriguing findings advance our current understanding of Spn interactions with its obligate human host.

### Hemoglobin facilitates vigorous growth in iron-depleted medium

The reinstatement of Spn growth in the iron-depleted medium, THYB-DP, by hemoglobin or heme (Fig. 1B,C) shows that Spn can metabolize heme iron, as it was previously reported^21,25,35^. Hemoglobin iron, however, appears particularly beneficial, triggering strong Spn growth surpassing that seen with free iron or heme alone (Fig. 1). This is the first report to describe the unusually robust growth of Spn upon hemoglobin supplementation in an otherwise iron-deplete medium. Why had other investigators not seen this exquisite hemoglobin-specific enhanced growth? Technical differences, such as the use of the chelating resin, Chelex-100, might have masked the positive impact of hemoglobin in prior investigations^21,25,35^. Chelex-100 has broad specificity, and treatment with this resin might have limited the availability of additional cations that are needed for full Spn growth. Furthermore, the long pre-cultivation in iron-deprived medium prior to the addition of hemoglobin used in some studies^21,25,35^, might also have limited maximal growth with hemoglobin.

THYB-DP supplemented with heme above 5–10 μM (equivalent to 20–40 μM hemoglobin) was growth inhibitory. In excess, heme is harmful to many bacteria, including the related *Streptococcus pyogenes*, due to its lipophilic and oxidative nature, which damages the bacterial membranes, proteins, and nucleic acids^40,41^. The negative impact that is inherent to the presence of free heme in the medium may prevent the benefit of heme that is provided by hemoglobin. Hemoglobin likely delivers the heme directly to surface receptors and membrane transporters for import^24,36,42^. Compared to free heme, heme that is delivered from one protein to another likely has fewer opportunities to damage the cell envelope.

### Spn growth stimulation is hemoglobin-dependent and utilizes a unique mechanism

Supplementing THYB with hemoglobin enhances pneumococcal growth, shortening the lag period, and increasing maximal biomass and viability (Fig. 2A,B and Table 1). Other host proteins, inactivated hemoglobin, or the flow-through collected after hemoglobin filtration, did not have this effect. Hence, it is hemoglobin in its native form, rather than a nonspecific increase in peptides and amino acids availability, or the presence of low molecular weight contaminants, that is advantageous for Spn growth. Spn is often grown with a source of catalase (e.g., blood) to neutralize a large amount of hydrogen peroxide this bacterium can produce. If inhibition of H_2_O_2_ by hemoglobin allowed enhanced growth, then the addition of catalase to THYB would have improved growth, but this did not occur (Fig. 2C), suggesting that hemoglobin does not extend growth by scavenging the H_2_O_2_ from the medium.

Supplementation with ferric iron did not enhance Spn growth in THYB, suggesting that the iron levels in this medium are not growth-limiting for Spn (Fig. 2D). Moreover, the cellular levels of iron in cells grown in THYB with hemoglobin are similar to those found in cells grown in regular THYB (Fig. 2F). Therefore, hemoglobin growth benefits are not derived by facilitating an increase in the total iron level in Spn. Hemoglobin, however, might have increased the cellular heme-to-iron ratio compared with cells that grew in THYB. Hence, we speculate that hemoglobin promotes growth, at least in part, by donating heme and that heme is more growth beneficial then metal iron. The finding that Spn imports less of the metal when THYB is enriched with ferric iron (Fig. 2F) supports the idea that heme might be more useful for the pathogen's physiology than free iron. Other pathogenic bacteria, such as *Staphylococcus aureus* and *S. pyogenes,* demonstrate a preference for heme when supplied with both free iron and heme^43,44^. Canonical heme degradation results in the production of biliverdin and its reduced product bilirubin^45^, both molecules scavenge various ROS and are considered potent antioxidants^46,47^. Perhaps, similar to the human host, heme catabolism by Spn leads to the production of protective end products and thus is valuable beyond iron release. Studies in our laboratories are underway to address this hypothesis.

The positive response to hemoglobin was rigorously assessed in the current study and found to be shared by multiple pneumococcal strains and independent of the growth assay used (Fig. 3). The addition of hemoglobin shortens the lag period in THYB inoculated with overnight cultures that grew either on BAPs or in THYB (Fig. 3B). Nevertheless, cells that were collected from BAPs exhibited improved growth. It's possible that the presence of hemoglobin is one of the growth-promoting factors in BAPs, which are commonly used for Spn cultivation. Notably, the addition of hemoglobin to THYB inoculated with logarithmic cells still enhanced growth (Fig. 3B). Hence, hemoglobin acts by both stimulating growth in resting cells and by extending the growth period before an exponentially growing culture enter the stationary phase. Spn growth with hemoglobin appeared biphasic in all conditions, other than in cultures inoculated with logarithmic cells. The formation of a two-step growth curve was more apparent in the experiments using Spn grown on THYB as the inoculum (Fig. 3B), in CAT medium (Fig. 3C), and with the Δ*spbhp-37* mutant (Fig. 8A,B). We have not explored in detail this biphasic growth pattern. Still, the absence of the first growth phase in cultures using logarithmic cultures implies that the initial hemoglobin impact on resting cells can be separated from its influence on exponentially growing cells.

It is not fully understood why Spn exhibits limited growth in batch cultures and enters early into the stationary phase. Acidification and other growth conditions were implicated as growth limiting factors. Nevertheless, at least some Spn strains (such as D39), still demonstrated limited exponential growth even under conditions where the pH and other factors such as temperature and atmosphere were controlled^10,48^. The data here show that hemoglobin extends Spn growth in medium that was not otherwise replenished, without pH control, with or without pre-cultivation. Therefore, it's possible that hemoglobin (directly and/or via the heme it donates) redirects a regulatory mechanism(s) that otherwise leads to premature growth arrest during in vitro cultivation. Interestingly, hemoglobin did not influence the expression of *lytA* and *spxB,* which cause Spn death, but the expression of these two genes was high under our experimental conditions (Fig. 5C). It is worth noting that for our transcriptome studies, hemoglobin was added at the early exponential growth, and gene expression was analyzed one and two hours after treatment; thus, we can't rule out a later change in *lytA* and *spxB* expression.

### Hemoglobin has a broad impact on Spn transcriptome suggesting host adaptation

Hemoglobin triggered significant transcriptome remodeling (Fig. 4). Various genes with an established role in colonization were activated (Fig. 5A), including the *aliA/aliB* genes. The lipoproteins, AliA, and AliB, are paralogs of AmiA, the oligopeptide-binding component of the Ami system, which is used by the auxotroph Spn to import oligopeptides^49^. Inactivation of *ami-aliA/aliB* attenuates adherence to pulmonary epithelial cells^50^, and these proteins are important for the initial colonization of the nasopharynx^31^. Hemoglobin also induced the PGN hydrolase gene, *lytB,* whose activity is necessary for the adherence and invasion of human lung epithelial cells^32^. The colonizing factor PavB also positively responded to hemoglobin. PavB contributes to nasopharyngeal colonization and is highly expressed during heart infection^34,51^. Similarly, hemoglobin induced the arginine biosynthesis genes, *argGH*, which are needed for growth in the lung, blood, and cerebrospinal fluids^52^. Hence, Spn transcriptome response to hemoglobin suggests the pathogen perceives the presence of hemoglobin as a cue for the host environment.

### Hemoglobin activates pneumococcal utilization of glycoproteins found in the host mucin and the epithelial mucosa

Hemoglobin had a strong influence on the expression of many genes involved in the uptake and use of host-derived sugars found in the mucosa and extracellular matrix (Fig. 6). Spn can ferment about 32 different carbohydrates in vitro^38^, and over 30% of the transporters in the Spn genome are dedicated to carbohydrate uptake^53^, many of which contribute to colonization and pathogenesis. Free sugars, however, are not typically available in the upper respiratory tract^54,55^; rather, they are linked to the glycoproteins (i.e., O- and N-linked glycan and glycosaminoglycan) found on the epithelial cell lining and in mucin^56,57^. Sequential deglycosylation by various pneumococcal exo-glycosidases (e.g., BgaA, BgaC, NanA, NanB, and StrH) allow Spn to cleave the sugars from the host glycan for uptake (^57–59^ and Fig. 7A). Our transcriptome analysis suggests that hemoglobin activates the expression of genes needed for the release, import, or catabolism of the host glycan derived monosaccharides, galactose and mannose, glycosaminoglycan disaccharides (e.g., hyaluronic acid), and the beta-glucosides amygdalin and cellobiose (Fig. 6A). Therefore, like mucin^60^, hemoglobin induces the expression of Spn genes required for growth on sugars derived from the host glycans. In vitro growth assays confirmed that Spn could use the human α − 1-acid glycoprotein AGP (N-linked glycan) as a carbohydrate source when grown in the presence of hemoglobin (Fig. 7B), but not in the presence of human serum albumin, HSA, as a control (Fig. 7C). Hemoglobin down-regulated the operons encoding the glycerol MIP family transporter, *glp,* and the raffinose transporter (Fig. 6B). Interestingly, Spn ear isolates demonstrate a reduced capacity to use the sugar raffinose comparing with blood isolates^61^.

### The hemoglobin/heme receptor Spbhp-3*7* has a role in mediating the hemoglobin response and growth benefits

PiuA and Spbhp-3*7* are the only two hemoglobin (and heme) receptors with an established role in heme uptake. Hemoglobin, however, represses expression of the *piuBCDA* transporter, while the expression of *spbhp-37* remained high whether hemoglobin was added or not (more than 30-fold above *piuA*, Fig. 5C). Using a *Δspbhp-37* mutant, we showed that Spbhp-37 plays an important role in the use of heme and hemoglobin iron (Fig. 8A). This is consistent with the observation that inhibition of Spbhp-37 by antibody interfered with Spn growth on hemoglobin iron^25^. Spn, however, was still able to utilize hemoglobin, likely due to redundancy in heme uptake systems. The Δ*spbhp-37* mutant did not grow well in THYB, the addition of hemoglobin improved growth only partially, and lower iron levels were found in mutant cells grown in THYB with hemoglobin (Fig. 8C). These observations suggest that Spbhp-37 has a redundant role in mediating the growth benefit of hemoglobin, possibly by capturing heme from hemoglobin. Notably, hemoglobin failed to induce the expression of the *aliA* and *argG* genes in the Δ*spbhp-37* mutant as it did in the wildtype strain (Fig. 8D). Hence, *spbhp-37* contributes to the shift in gene expression caused by hemoglobin. Further investigations are needed to determine if Spbhp-37 influence on gene expression depends on its role in heme import.

Spn is listed as a severe threat in the 2019 CDC antimicrobial-resistant (AMR) report^62^. Therefore, it is critical to gain insights into Spn pathophysiology to better design new treatment modalities. Herein, we have demonstrated that hemoglobin greatly benefits pneumococcal cultivation in vitro, promoting growth and viability, and causes a transcriptome shift that is likely to advance Spn preparation for colonization and infection. Hence, this study makes significant contributions to the understanding of pneumococcal growth requirements and adaptation to its obligate human host. Additional work is needed to describe the mechanism by which Spn perceives and responds to hemoglobin and the effect of hemoglobin on Spn during infection.

## Experimental procedures

### Bacterial strains and growth media

The Spn strains used in this study are listed in Table 2. Frozen Spn stocks were prepared in the medium Skim milk-Tryptone-Glucose-Glycerin (STGG) as described^63^ and kept at -80 °C. Spn STGG stocks were plated on Tryptic Soy blood agar plates (BAPs) and incubated overnight at 37 °C under microaerophilic conditions. Spn was also grown in Todd-Hewitt broth containing 0.5% (w/vol) yeast extract (THYB) or in Casein-Tryptone (CAT) medium^64^ containing Bacto Casitone 10 g/l, Bacto Tryptone 10 g/l, Yeast Extract 1 g/l, NaCl 5 g/l, 0.5 M K_2_HPO_4_ (30 ml/l) and 200 U/µl of catalase. One or more of the following supplements were added to the growth medium as indicated: The iron chelator 2, 2′-Dipyridyl (ACROS Organics), bovine hemin (Sigma Aldrich), human hemoglobin (Sigma Aldrich), Ferric nitrate nonahydrate (FeNO_3_, Thermo Fisher Scientific), bovine serum albumin (BSA, Sigma Aldrich), 200 U/µl of catalase (Sigma Aldrich), glucose (Sigma Aldrich), human α − 1 acid glycoprotein (AGP, Sigma Aldrich), or fatty-acid free human serum albumin (Sigma Aldrich). Some experiments used frozen logarithmic Spn stocks as the starting inoculum. To prepare such stocks, Spn cells growing in THYB were collected at the early logarithmic phase of growth (O.D._600_ = 0.2–0.3), and glycerol was added to the culture to a final 10% (vol/vol) and stored at − 80 °C.

**Table 2:** Strains used in this study.

*S. pneumoniae*	Description	Source or references
D39	Avery strain, clinical isolate, WT (capsular serotype 2), CSP1	^72,73^
TIGR4	Invasive clinical isolate, WT (capsular serotype 4), CSP2	^53^
8,655	Invasive isolate (serotype 6B), CSP2	CDC
3,875	Invasive isolate (serotype 6B), CSP1	CDC
∆*spbhp-37* (SPZE1)	D39-derivative *spbhp-37* null mutant, Ery^r^	This study
***E. coli***		
One shot Top-10	Cloning strain	Invitrogen
**Plasmids**		
pAF104	Seamless cloning vector pUC19, Amp^r^	This study
pCR2.1 TOPO	Cloning vector, Ery^r^	^67^

### Growth assays

Fresh medium (with or without supplements) was inoculated with Spn cells collected from BAPs following overnight incubation (starting O.D._600_ = 0.05) or from frozen logarithmic culture (starting culture O.D._600_ = 0.02) as indicated. Cell cultures (200 μl per well) were allowed to grow in 96-well microtiter plates (Corning) incubated at 37 °C. The culture O.D._600_ was recorded at 1 h intervals for 18 h using a SpectraMax M2 spectrophotometer (Molecular Device). For each growth condition, we used wells containing only the medium (and supplements when appropriate) as the blank. Bacterial growth was tested in triplicates. To determine cell viability, culture samples were collected at designated time points, serially diluted in 0.9% saline, and plated in triplicates into BAP.

### Total iron determination by ICP-MS

Fresh THYB medium containing 80 µM FeNO_3_ or 20 µM hemoglobin was inoculated with Spn grown on BAPs (starting O.D._600_ = 0.05) and incubated at 37 °C for 6 h in 12 well microtiter plates. Culture samples (6 ml, O.D._600_ = 1) were washed three times with phosphate-buffered saline (PBS) prior to collection. The cell pellet was digested and analyzed (Center for Applied Isotope Studies, University of Georgia, Athens, GA) as described^65,66^.

### Construction of ∆*spbhp-37* mutant

The plasmids used in this study are listed in Table 2 and the primers in Table 3. We cloned a Δ*spbhp-37* mutant in Spn strain D39 by replacing the *spbhp-37* coding sequence with that of *ermB* (erythromycin resistance^67^), such that the *ermB* ORF is under the transcriptional control of *spbhp-37* promoter and terminator. The mutant allele containing *ermB* ORF flanked by the 5′ and 3′ genomic regions of the *spbhp-37* gene was prepared using the Gene art seamless cloning kit (Thermo fisher scientific). Briefly, the appropriate genomic segments were amplified from D39 chromosome using the primer sets ZE 740-L/ZE 741-R and ZE 744-L/ZE 745-R. The *ermB* gene and the pUC19 vector were amplified from pCR2.1 topo and pUC19-L plasmids using the primer sets ZE 742-L/ZE 743-R and ZE 738-L/ZE 739-R respectively. All PCR fragments were purified (using the MinElute PCR Purification Kit, Qiagen) and cloned into One Shot TOP10 *E. coli* strain, generating plasmid pAF104. The resulting allele was then amplified (from pAF104) and transformed into competent D39 cells using standard protocols^68^. The mutants were selected on BAPs containing erythromycin (0.5 µg/ml). The mutation was confirmed by PCR in the resulting erythromycin-resistant clones using the primer set ZE 740-L/ZE 745-R. qRT-PCR analysis confirmed that the Δ*spbhp-37* mutation did not change the expression of the downstream gene, SPD_0740 (Fig. S2).

**Table 3:** Primers used in this study.

Target	Primers	Sequence (5′–3′)	Comments
*pUC19-L*	ZE 738-L	TATCAAAGGGCATGCAAGCTTGGCGTAATCAT	Cloning
	ZE 739-R	ACTGTGCAGTACCGAGCTCGAATTCACTGGCC	
*5′-region of spbhp 37*	ZE 740-L	CTCGGTACTGCACAGTAGTAGGTTTCCCTTTG	Cloning
	ZE 741-R	TTGTTCATTACTGAACCTCCTAAATAAGATGT	
*ermB*	ZE 742-L	GTTCAGTAATGAACAAAAATATAAAATATTCT	Cloning
	ZE 743-R	CATCAAGGCGACTCATAGAATTATTTCCTCCC	
*3′-region of spbhp 37*	ZE 744-L	ATGAGTCGCCTTGATGGAAGCGTAAAAGTTCC	Cloning
	ZE 745-R	TGCATGCCCTTTGATAGACAAAACCACTTCTT	
*aliB*	ZE 878-L	GGACTGTTTCTCAGGACGGTTTG	qPCR
	ZE 879-R	CAGCTGCATATTGCAAACCTGTC	
*ilvD*	ZE 880-L	CCTGGTATGCGTTTCTCTCTAAC	qPCR
	ZE 881-R	AGCAATCATAGATCCAGGCATG	
*aliA*	ZE 882-L	GGTCACTTATGGGGATGAATGG	qPCR
	ZE 883-R	GGAATGTCACACCTTCTGCTTG	
*argG*	ZE 884-L	CCTTGGTATCTGCCTTGAGC	qPCR
	ZE 885-R	GATCCAAGGCTGCAATCGATAC	
*SPD_1803*	ZE 886-L	GGATTGGATGAGGATTTCTACC	qPCR
	ZE 887-R	CTTCTCTAACAAGCCAAGACATG	
*piuB*	ZE 864-L	TGATTTCGACCAGCAGACCTG	qPCR
	ZE 865-R	CTGTACTCGGTGCAGCAAACTG	
*rafE*	ZE 964-L	GCTTGCTCCAACTATGGTAAATC	qPCR
	ZE 965-R	CATTGACGACTTTGACCTTGATC	
*brnQ*	ZE 966-L	CTCTATCTGGAGAACATTTTCTTCC	qPCR
	ZE 967-R	GCTATCTTCGTTGAAATCTCGTAG	
*SPD_0740*	ZE 962-L	GTCATTGAGATGCGTGATATTACC	qPCR
	ZE 963-R	CATGTTCATTAGCGTGGACTTAC	
*gyrB*	ZE 661-L	GGCACTGTATGGTATCACACAAG	qPCR
	ZE 662-R	TCTCTAAATTGGGAGCGAATGTC	

### Spn growth with human α − 1 acid glycoprotein and serum albumin

To remove contaminating free sugars, human α − 1 acid glycoprotein (AGP, 10 mg/ml) was dialyzed in water using Slide-A-Lyzer G2 Dialysis Cassette (10 K MWCO, Thermo Fisher Scientific) as described^58^. The samples were then concentrated with Amicon Ultra-0.5 mL Centrifugal Filter Unit (10 K MWCO, Millipore Sigma), reconstituted in CAT medium (5 mg/ml) and filter sterilized. Spn D39 grown overnight on BAP were collected, washed and suspended in CAT. These cell suspensions were used to inoculate fresh CAT medium with or without glucose (0.5% (w/v), AGP (5 mg/ml) or HSA (20 μM). Bacterial growth in the presence or absence of 20 μM hemoglobin in 96-well microtiter plates was monitored as described above.

### RNA-Seq analysis

Fresh THYB was inoculated with Spn cells from frozen logarithmic stocks (starting culture O.D._600_ = 0.02) and the cultures were allowed to grow in 12 well microtiter plates (2 ml per well) at 37 °C. 20 µM hemoglobin (in 0.9% saline) or 0.9% saline (negative control) was added to the growing cells at the early logarithmic phase (O.D._600_ = 0.2–0.3). Cultures samples (four biological replicates for each condition) were collected and mixed with RNA protect reagent (Qiagen) following the manufacturer's recommendations. Cells were then collected by centrifugation and stored at -80 °C. For RNA preparation, cell samples were suspended in 700 μl of Trizol with 300 mg of acid-washed glass beads (Sigma Life Science) and disrupted by vortexing. Total RNA was isolated using the Direct-zol RNA MiniPrep kit (Zymo Research). DNA was removed using the Turbo DNase-free kit (Life Technologies). rRNA was eliminated with the Ribo-Zero Magnetic kit for Gram-positive bacteria (Epicenter). RNA Quality and quantity were assessed using a 2,100 Bioanalyzer (Agilent) and NanoDrop 8,000 spectrophotometer (Thermo Fisher Scientific), respectively. Directional RNA-Seq libraries were created using the ScriptSeq v2 RNA-Seq Library Preparation kit (Illumina) according to the manufacturer's instructions. A rapid-run 100 bp single-read DNA sequencing was performed at the Institute for Bioscience and Biotechnology Research (IBBR) Sequencing Facility at the University of Maryland, College Park, using the Illumina HiSeq 1,500 platform. Data were generated in the standard Sanger FastQ format and raw reads were deposited with the Sequence Read Archive (SRA) at the National Center for Biotechnology Institute (accession PRJNA626052). Read quality was evaluated using FastQC software, and mapping against the Spn D39 genome using Bowtie package alignment software^69^. The read count or raw count data for all genes were acquired using Feature count package^70^. These raw count data files were then used in DESeq2 package^71^ to calculate differential expression analysis of all samples for pairwise comparison. Graph Pad Prism (version 8.3.1) was used to prepare dot plot representation of gene expression levels using normalized RNA-seq read counts.

### qRT-PCR analysis

Quantitative reverse transcription PCR (qRT-PCR) analysis was carried out using the Power SYBR Green RNA-to-Ct 1-Step Kit (Applied Biosystems) and 7,500 Fast Real-Time PCR machine (Applied Biosystems) according to the manufacturer's specifications. A total of 25 ng RNA was used per qRT-PCR reactions and each reaction was done in duplicates. Primers used for qRT-PCR are listed in Table 3. The relative expression was normalized to the endogenous control *gyrB* gene and fold changes were calculated using the comparative 2^−ΔΔCT^ method.

## Supplementary information

Supplementary Information.

## Data Availability

The authors have deposited all RNA-seq raw sequencing reads with the Sequence Read Archive (SRA) at the National Center for Biotechnology Institute (accession PRJNA626052) for public availability.

## References

[1] Eurich DT, Marrie TJ, Minhas-Sandhu JK, Majumdar SR (2017). Risk of heart failure after community acquired pneumonia: prospective controlled study with 10 years of follow-up. BMJ.

[2] Musher DM, Rueda AM, Kaka AS, Mapara SM (2007). The association between pneumococcal pneumonia and acute cardiac events. Clin. Infect. Dis..

[3] Levine OS, Klugman KP (2009). Editorial: breathing new life into pneumonia epidemiology. Am. J. Epidemiol..

[4] Walker CLF (2013). Global burden of childhood pneumonia and diarrhoea. Lancet.

[5] Kadioglu A, Weiser JN, Paton JC, Andrew PW (2008). The role of Streptococcus pneumoniae virulence factors in host respiratory colonization and disease. Nat. Rev. Microbiol..

[6] Weiser JN, Ferreira DM, Paton JC (2018). Streptococcus pneumoniae: transmission, colonization and invasion. Nat. Rev. Microbiol..

[7] Jedrzejas MJ (2001). Pneumococcal virulence factors: structure and function. Microbiol. Mol. Biol. Rev..

[8] Klugman KP, Madhi SA, Albrich WC (2008). Novel approaches to the identification of *Streptococcus pneumoniae* as the cause of community-acquired pneumonia. Clin. Infect. Dis..

[9] van der Poll T, Opal SM (2009). Pathogenesis, treatment, and prevention of pneumococcal pneumonia. Lancet.

[10] Carvalho SM, Kuipers OP, Neves AR (2013). Environmental and nutritional factors that affect growth and metabolism of the pneumococcal serotype 2 strain D39 and its nonencapsulated derivative strain R6. PLoS ONE.

[11] Goncalves VM (2003). Purification of capsular polysaccharide from *Streptococcus pneumoniae* serotype 23F by a procedure suitable for scale-up. Biotechnol. Appl. Biochem..

[12] Massaldi H (2010). Features of bacterial growth and polysaccharide production of *Streptococcus pneumoniae* serotype 14. Biotechnol. Appl. Biochem..

[13] Slotved HC, Satzke C (2013). In vitro growth of pneumococcal isolates representing 23 different serotypes. BMC Res. Notes.

[14] Brown JS, Gilliland SM, Ruiz-Albert J, Holden DW (2002). Characterization of pit, a *Streptococcus pneumoniae* iron uptake ABC transporter. Infect. Immun..

[15] Berry AM, Lock RA, Hansman D, Paton JC (1989). Contribution of autolysin to virulence of *Streptococcus pneumoniae*. Infect. Immun..

[16] Berry AM, Paton JC (2000). Additive attenuation of virulence of *Streptococcus pneumoniae* by mutation of the genes encoding pneumolysin and other putative pneumococcal virulence proteins. Infect. Immun..

[17] Regev-Yochay G, Trzcinski K, Thompson CM, Lipsitch M, Malley R (2007). SpxB is a suicide gene of *Streptococcus pneumoniae* and confers a selective advantage in an in vivo competitive colonization model. J. Bacteriol..

[18] 18Grousd, J. A., Rich, H. E. & Alcorn, J. F. Host-pathogen interactions in gram-positive bacterial pneumonia. *Clin. Microbiol. Rev. 32* (2019). 10.1128/CMR.00107-18 PMC6589866 31142498

[19] Lopez CA, Skaar EP (2018). The impact of dietary transition metals on host-bacterial interactions. Cell Host Microbe.

[20] Palmer LD, Skaar EP (2016). Transition metals and virulence in bacteria. Annu. Rev. Genet..

[21] Tai SS, Lee CJ, Winter RE (1993). Hemin utilization is related to virulence of *Streptococcus pneumoniae*. Infect. Immun..

[22] Turner AG, Ong CY, Walker MJ, Djoko KY, McEwan AG (2017). Transition metal homeostasis in *Streptococcus pyogenes* and *Streptococcus pneumoniae*. Adv. Microb. Physiol..

[23] Ge R, Sun X (2014). Iron acquisition and regulation systems in Streptococcus species. Metallomics.

[24] Tai SS, Yu C, Lee JK (2003). A solute binding protein of *Streptococcus pneumoniae* iron transport. FEMS Microbiol. Lett..

[25] Romero-Espejel ME, Rodriguez MA, Chavez-Munguia B, Rios-Castro E, Olivares-Trejo Jde J (2016). Characterization of Spbhp-37, a hemoglobin-binding protein of *Streptococcus pneumoniae*. Front. Cell Infect. Microbiol..

[26] Miao X (2018). A novel iron transporter SPD_1590 in *Streptococcus pneumoniae* contributing to bacterial virulence properties. Front. Microbiol..

[27] Cook, L. C. C. *et al.* Transcriptomic analysis of *Streptococcus pyogenes* colonizing the vaginal mucosa identifies hupY, an MtsR-regulated adhesin involved in heme utilization. *mBio* **10** (2019). 10.1128/mBio.00848-19 PMC6593403 31239377

[28] Ponka P, Grady RW, Wilczynska A, Schulman HM (1984). The effect of various chelating agents on the mobilization of iron from reticulocytes in the presence and absence of pyridoxal isonicotinoyl hydrazone. Biochem. Biophys. Acta..

[29] King KY, Horenstein JA, Caparon MG (2000). Aerotolerance and peroxide resistance in peroxidase and PerR mutants of *Streptococcus pyogenes*. J. Bacteriol..

[30] Bates CS, Montanez GE, Woods CR, Vincent RM, Eichenbaum Z (2003). Identification and characterization of a *Streptococcus pyogenes* operon involved in binding of hemoproteins and acquisition of iron. Infect. Immun..

[31] Kerr AR (2004). The Ami-AliA/AliB permease of *Streptococcus pneumoniae* is involved in nasopharyngeal colonization but not in invasive disease. Infect. Immun..

[32] Bai XH (2014). Structure of pneumococcal peptidoglycan hydrolase LytB reveals insights into the bacterial cell wall remodeling and pathogenesis. J. Biol. Chem..

[33] Eijkelkamp BA (2016). The first histidine triad motif of PhtD Is critical for zinc homeostasis in *Streptococcus pneumoniae*. Infect. Immun..

[34] Jensch I (2010). PavB is a surface-exposed adhesin of *Streptococcus pneumoniae* contributing to nasopharyngeal colonization and airways infections. Mol. Microbiol..

[35] Brown JS, Gilliland SM, Holden DW (2001). A *Streptococcus pneumoniae* pathogenicity island encoding an ABC transporter involved in iron uptake and virulence. Mol. Microbiol..

[36] Romero-Espejel ME, Gonzalez-Lopez MA, Olivares-Trejo Jde J (2013). Streptococcus pneumoniae requires iron for its viability and expresses two membrane proteins that bind haemoglobin and haem. Metallomics.

[37] Shafeeq S, Kuipers OP, Kloosterman TG (2013). Cellobiose-mediated gene expression in *Streptococcus pneumoniae*: a repressor function of the novel GntR-type regulator BguR. PLoS ONE.

[38] Bidossi A (2012). A functional genomics approach to establish the complement of carbohydrate transporters in *Streptococcus pneumoniae*. PLoS ONE.

[39] Dehal PS (2010). MicrobesOnline: an integrated portal for comparative and functional genomics. Nucl. Acids Res..

[40] Anzaldi LL, Skaar EP (2010). Overcoming the heme paradox: heme toxicity and tolerance in bacterial pathogens. Infect. Immun..

[41] Sachla AJ, Le Breton Y, Akhter F, McIver KS, Eichenbaum Z (2014). The crimson conundrum: heme toxicity and tolerance in GAS. Front. Cell Infect. Microbiol..

[42] Sheldon JR, Heinrichs DE (2015). Recent developments in understanding the iron acquisition strategies of gram positive pathogens. FEMS Microbiol. Rev..

[43] Montañez GE, Neely MN, Eichenbaum Z (2005). The streptococcal iron uptake (Siu) transporter is required for iron uptake and virulence in a zebrafish infection model. Microbiology (Reading, England).

[44] Rouault TA (2004). Microbiology. Pathogenic bacteria prefer heme. Science (New York, N.Y.).

[45] Lyles KV, Eichenbaum Z (2018). From host heme to iron: the expanding spectrum of heme degrading enzymes used by pathogenic bacteria. Front. Cell Infect. Microbiol..

[46] Foresti, R., Green, C. J. & Motterlini, R. Generation of bile pigments by haem oxygenase: a refined cellular strategy in response to stressful insults. *Biochem. Soc. Symp.* 177–192 (2004). 10.1042/bss0710177 15777021

[47] Vitek L, Schwertner HA (2007). The heme catabolic pathway and its protective effects on oxidative stress-mediated diseases. Adv. Clin. Chem..

[48] Mercade M, Lindley ND, Loubiere P (2000). Metabolism of *Lactococcus lactis* subsp. cremoris MG 1363 in acid stress conditions. Int. J. Food Microbiol..

[49] Claverys JP, Grossiord B, Alloing G (2000). Is the Ami-AliA/B oligopeptide permease of *Streptococcus pneumoniae* involved in sensing environmental conditions?. Res. Microbiol..

[50] Cundell DR, Pearce BJ, Sandros J, Naughton AM, Masure HR (1995). *Peptide permeases* from *Streptococcus pneumoniae* affect adherence to eucaryotic cells. Infect. Immun..

[51] Shenoy AT (2017). *Streptococcus pneumoniae* in the heart subvert the host response through biofilm-mediated resident macrophage killing. PLoS Pathog..

[52] Piet JR (2014). *Streptococcus pneumoniae* arginine synthesis genes promote growth and virulence in pneumococcal meningitis. J. Infect. Dis..

[53] Tettelin H (2001). Complete genome sequence of a virulent isolate of *Streptococcus pneumoniae*. Science (New York, N.Y.).

[54] Philips BJ, Meguer JX, Redman J, Baker EH (2003). Factors determining the appearance of glucose in upper and lower respiratory tract secretions. Intensive Care Med..

[55] Shelburne SA, Davenport MT, Keith DB, Musser JM (2008). The role of complex carbohydrate catabolism in the pathogenesis of invasive streptococci. Trends Microbiol.

[56] Rose MC, Voynow JA (2006). Respiratory tract mucin genes and mucin glycoproteins in health and disease. Physiol. Rev..

[57] Yesilkaya H, Manco S, Kadioglu A, Terra VS, Andrew PW (2008). The ability to utilize mucin affects the regulation of virulence gene expression in *Streptococcus pneumoniae*. FEMS Microbiol. Lett..

[58] Burnaugh AM, Frantz LJ, King SJ (2008). Growth of *Streptococcus pneumoniae* on human glycoconjugates is dependent upon the sequential activity of bacterial exoglycosidases. J. Bacteriol..

[59] Terra VS, Homer KA, Rao SG, Andrew PW, Yesilkaya H (2010). Characterization of novel beta-galactosidase activity that contributes to glycoprotein degradation and virulence in *Streptococcus pneumoniae*. Infect. Immun..

[60] Paixao L (2015). Host glycan sugar-specific pathways in *Streptococcus pneumoniae*: galactose as a key sugar in colonisation and infection [corrected]. PLoS ONE.

[61] Minhas, V. *et al.* Capacity to utilize raffinose dictates pneumococcal disease phenotype. *MBio* **10** (2019). 10.1128/mBio.02596-18 PMC6336424 30647157

[62] CDC. Antibiotic resistance threats in the United States (2019).

[63] O'Brien KL (2001). Evaluation of a medium (STGG) for transport and optimal recovery of *Streptococcus pneumoniae* from nasopharyngeal secretions collected during field studies. J. Clin. Microbiol..

[64] Porter RD, Guild WR (1976). Characterization of some pneumococcal bacteriophages. J. Virol..

[65] EPA, U. S. Vol. Revision 5.4 (1994).

[66] EPA, U. S. Method 3052: Microwave Assisted Acid Digestion of Siliceous and Organically Based Matrices. **Revision 0** (1996).

[67] Vidal JE, Ludewick HP, Kunkel RM, Zahner D, Klugman KP (2011). The LuxS-dependent quorum-sensing system regulates early biofilm formation by *Streptococcus pneumoniae* strain D39. Infect. Immun..

[68] Havarstein LS, Coomaraswamy G, Morrison DA (1995). An unmodified heptadecapeptide pheromone induces competence for genetic transformation in *Streptococcus pneumoniae*. Proc. Natl. Acad. Sci. USA.

[69] Langmead B, Trapnell C, Pop M, Salzberg SL (2009). Ultrafast and memory-efficient alignment of short DNA sequences to the human genome. Genome Biol..

[70] Liao Y, Smyth GK, Shi W (2014). featureCounts: an efficient general purpose program for assigning sequence reads to genomic features. Bioinformatics.

[71] Love MI, Huber W, Anders S (2014). Moderated estimation of fold change and dispersion for RNA-seq data with DESeq2. Genome Biol..

[72] Avery OT, Macleod CM, McCarty M (1944). Studies on the chemical nature of the substance inducing transformation of pneumococcal types : induction of transformation by a desoxyribonucleic acid fraction isolated from pneumococcus type III. J. Exp. Med..

[73] Lanie JA (2007). Genome sequence of Avery's virulent serotype 2 strain D39 of *Streptococcus pneumoniae* and comparison with that of unencapsulated laboratory strain R6. J. Bacteriol..

