# Supplementary material for: Hemoglobin stimulates vigorous growth of Streptococcus pneumoniae and shapes the pathogen's global transcriptome
Source: Sci Rep. 2020 Sep 16;10:15202. doi: 10.1038/s41598-020-71910-1 (PMC7494912; doi:10.1038/s41598-020-71910-1)
Supplement: Supplementary file 1 — Supplementary Information. [file 41598_2020_71910_MOESM1_ESM.pdf]

Supplementary Materials for:

Hemoglobin stimulates vigorous growth of *Streptococcus pneumoniae* and shapes the pathogen's global transcriptome

Fahmina Akhter[1], Edroyal Womack[1], Jorge E. Vidal[2], Yoann Le Breton[3, 4], Kevin S. McIver[3], Shrikant Pawar[1, 5] and Zehava Eichenbaum*[1]

[1]Department of Biology, Georgia State University, Atlanta, Georgia, USA.

[2]Department of Microbiology and Immunology, University of Mississippi Medical Center, Jackson, MS, USA

[3]Department of Cell Biology & Molecular Genetics, Maryland Pathogen Research Institute, University of Maryland, College Park (UMCP), College Park, Maryland, USA.

[4]Current Address: Wound Infections Dept., Bacterial Diseases Branch, The Walter Reed Army Institute of Research, Silver Spring, MD.

[5]Current Address: Yale center for genome analysis, Yale University, New Haven, CT, USA.

Correspondence and requests for materials should be addressed to Zehava Eichenbaum (email: zeichen@gsu.edu)

Supplementary Materials contents:

Supplemental Figures:

Figure S1: Correlation of gene expression by RNA-Seq and qRT-PCR.

Figure S2: The Δ*spbhp-37:: ermB* mutation does not affect the expression of the downstream SPD_0740 gene.

Supplemental Table S1: Genes in D39 differentially expressed in One- and two-hour post hemoglobin treatment compared to in THYB.

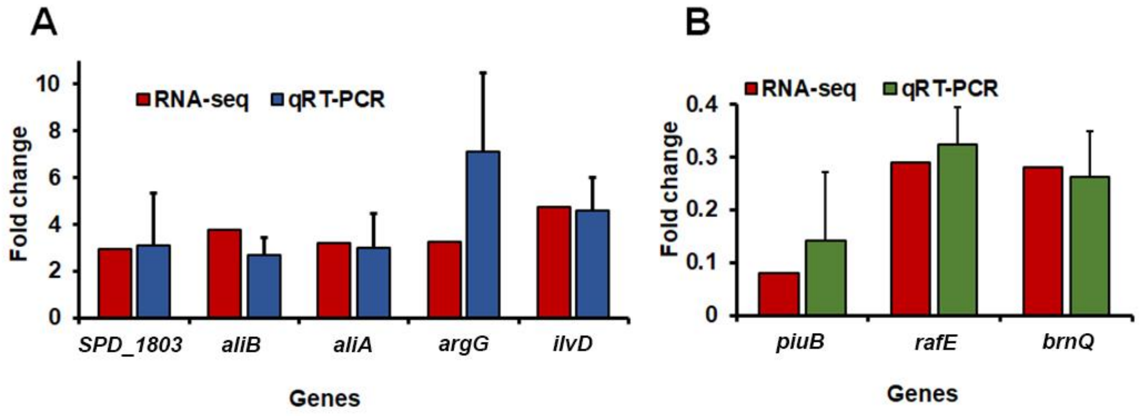

Figure S1: Correlation of gene expression by RNA-Seq and qRT-PCR. Fold change in the expression of selected genes 1 h post hemoglobin addition as determined by RNA-Seq and qRT-PCR. **A.** Up-regulated genes. **B.** Down-regulated genes. The experiments were performed in duplicates with at least two biological replicates. The replicates data are shown as the mean ± SD.

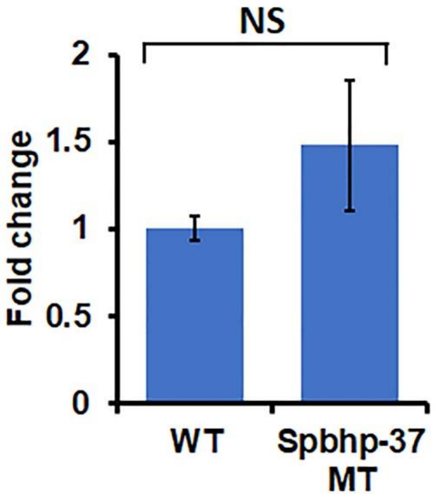

Figure S2: The Δ*spbhp-37:: ermB* mutation does not affect the expression of the downstream SPD_0740 gene. Fold change in the expression of SPD_0740 2 h post hemoglobin in the wildtype and mutant strains as determined by qRT-PCR. The experiments were performed in duplicates with at least two biological replicates. The replicates data are shown as the mean ± SD. The NS denotes no statistical significance, $P \leq 0.05$ (WT vs. MT, Student's t-test).

Supplemental Table S1. Genes in D39 differentially expressed in one- and two-hour post hemoglobin treatment compared to THYB

Gene ID	Gene	Fold change[a] (1h)	*p* value (1h)	Fold change[a] (2h)	*p* value (2h)
Up regulated					
SPD_0052		1.19	3.12E-01	2.64	2.25E-13
SPD_0053	*purF*	1.18	4.09E-01	2.07	1.78E-05
SPD_0058	*purD*	0.98	9.20E-01	2.77	1.74E-09
SPD_0059	*purE*	0.98	9.17E-01	2.70	2.76E-07
SPD_0060	*purK*	1.01	9.56E-01	2.90	1.91E-08
SPD_0065	*bgaC*	0.79	2.84E-01	2.06	7.28E-04
SPD_0067		1.43	2.42E-02	2.05	3.40E-04
SPD_0068		1.38	1.55E-01	2.37	6.42E-04
SPD_0070	*agaS*	1.22	2.34E-01	2.17	1.08E-04
SPD_0071	*galM*	1.23	3.19E-01	2.04	3.45E-03

SPD_0072		1.73	3.36E-03	8.88	5.22E-116
SPD_0073		0.71	1.05E-03	2.91	8.28E-21
SPD_0080	*pavB*	0.75	3.48E-01	2.92	3.27E-17
SPD_0109		4.62	1.38E-34	4.34	6.60E-06
SPD_0110	*argG*	3.27	3.45E-23	4.90	5.18E-06
SPD_0111	*argH*	3.87	1.67E-30	5.48	2.53E-07
SPD_0162		2.15	4.17E-05	4.57	4.23E-15
SPD_0163		2.97	1.07E-12	5.14	2.98E-43
SPD_0164		2.96	2.14E-06	5.70	9.03E-29
SPD_0192	*rpsJ*	2.00	7.63E-06	1.28	9.35E-03
SPD_0195	*rplW*	2.08	5.36E-10	1.84	1.42E-09
SPD_0236	*talC*	0.81	3.52E-01	2.12	1.78E-09
SPD_0293		0.88	6.43E-01	3.79	3.07E-07
SPD_0294		0.55	2.96E-02	2.06	4.94E-03
SPD_0295		0.67	1.32E-01	2.02	6.07E-03
SPD_0334	*aliA*	3.19	3.39E-10	3.28	4.56E-44
SPD_0335		1.51	1.33E-02	2.73	1.03E-14
SPD_0375		1.71	3.98E-09	3.81	1.53E-47
SPD_0376		1.53	7.78E-02	3.57	3.04E-32
SPD_0377		1.36	2.11E-01	2.11	4.88E-08
SPD_0383	*fabD*	1.25	4.00E-01	2.26	1.45E-03
SPD_0384	*fabG*	1.42	1.80E-01	2.31	6.85E-06
SPD_0385	*fabF*	1.28	3.44E-01	2.30	5.88E-06
SPD_0386	*accB*	1.35	2.36E-01	2.92	1.23E-09
SPD_0388	*accC*	1.28	3.49E-01	2.16	2.04E-05
SPD_0389	*accD*	1.47	1.30E-01	2.63	5.91E-08
SPD_0390	*accA*	1.58	6.00E-02	2.51	2.48E-07
SPD_0447	*glnR*	0.63	3.01E-02	1.00	9.66E-01
SPD_0448	*glnA*	0.64	2.77E-02	1.58	1.76E-06
SPD_0449		1.00	9.94E-01	3.33	9.25E-17
SPD_0521	*vex1*	1.55	2.09E-02	2.60	1.26E-13
SPD_0522	*vex2*	1.66	7.73E-03	2.53	3.91E-15
SPD_0523	*vex3*	1.50	1.81E-02	1.39	1.58E-01
SPD_0553	*secB*	2.66	1.95E-09	4.04	2.73E-14
SPD_0559		1.11	5.28E-01	3.17	1.17E-10
SPD_0560		1.02	9.06E-01	3.42	5.09E-06
SPD_0561		1.13	5.53E-01	2.95	1.77E-04
SPD_0562	*bgaA*	1.37	1.14E-01	1.14	6.33E-01
SPD_0592	*rsuA-2*	2.13	7.78E-04	1.66	3.35E-08
SPD_0608	*pyrF*	2.11	1.22E-03	1.94	1.72E-15
SPD_0609		2.28	2.65E-04	1.70	4.75E-07

SPD_0611		2.11	7.60E-15	1.55	3.64E-04
SPD_0612		2.37	6.39E-23	1.91	1.27E-07
SPD_0613		2.26	1.58E-10	1.61	7.16E-04
SPD_0614		2.07	5.57E-08	1.25	5.06E-01
SPD_0639		2.22	4.12E-02	2.36	1.08E-13
SPD_0648		1.37	1.35E-01	2.36	1.08E-13
SPD_0686		1.16	3.01E-01	2.35	1.12E-17
SPD_0687		1.27	8.73E-02	2.66	8.25E-17
SPD_0688		1.15	1.74E-01	2.46	3.34E-26
SPD_0753	*pcp*	1.83	5.04E-05	2.03	2.59E-09
SPD_0778		1.46	1.64E-01	2.53	7.12E-03
SPD_0803		2.71	1.59E-22	3.57	3.80E-45
SPD_0804		4.95	1.07E-72	2.56	7.49E-19
SPD_0805		4.65	3.00E-84	3.20	3.68E-36
SPD_0851	*pyrK*	2.65	1.46E-06	1.84	2.65E-11
SPD_0852	*pyrdb*	2.75	7.15E-07	1.70	7.08E-06
SPD_0853	*lytB*	2.37	4.70E-07	1.28	1.69E-02
SPD_0889	*phtD*	1.96	4.84E-04	2.05	1.09E-12
SPD_0900	*asd*	2.18	1.20E-05	1.63	4.04E-05
SPD_0901	*dapA*	2.01	1.03E-06	1.67	3.36E-06
SPD_0913		0.89	4.72E-01	2.38	1.92E-25
SPD_0977		1.22	1.45E-01	2.56	1.40E-15
SPD_1050	*lacD*	2.36	6.56E-05	5.30	2.26E-24
SPD_1051	*lacC*	2.11	6.57E-04	4.66	1.15E-22
SPD_1052	*lacB*	2.04	1.02E-03	4.00	3.47E-08
SPD_1053	*lacA*	1.90	2.95E-03	4.41	3.19E-22
SPD_1054		0.75	1.90E-01	2.26	1.94E-06
SPD_1057		0.87	4.86E-01	2.73	4.38E-08
SPD_1114		1.41	1.80E-01	2.07	7.50E-03
SPD_1187	*rplL*	1.59	2.06E-02	2.04	6.52E-08
SPD_1196	*mecA*	1.24	2.18E-01	2.28	1.34E-31
SPD_1226		2.66	7.57E-07	2.52	2.94E-15
SPD_1256		2.42	6.85E-08	2.66	8.37E-10
SPD_1257		2.27	2.28E-06	2.03	1.71E-05
SPD_1258		2.22	1.57E-06	2.02	1.10E-11
SPD_1300		2.08	1.63E-04	2.18	2.26E-06
SPD_1301		2.26	4.11E-07	4.16	1.99E-16
SPD_1302		2.35	2.36E-06	4.82	3.87E-32
SPD_1343		2.16	5.99E-06	1.47	9.49E-05
SPD_1344		2.41	5.01E-05	1.61	1.31E-02
SPD_1354		2.11	1.10E-02	4.74	1.57E-05

SPD_1355		4.14	4.11E-14	4.35	1.04E-11
SPD_1357	*aliB*	3.79	8.21E-16	5.78	3.41E-26
SPD_1372		1.50	3.91E-05	2.25	4.24E-15
SPD_1379		1.36	2.18E-01	2.93	6.26E-05
SPD_1380		1.48	6.36E-03	3.17	1.69E-29
SPD_1440		2.00	5.64E-04	5.47	1.31E-17
SPD_1568		2.01	1.27E-04	0.62	1.07E-03
SPD_1605		1.38	4.34E-05	2.29	5.25E-25
SPD_1611		1.20	5.37E-01	2.43	1.05E-03
SPD_1708		2.83	1.3E-18	1.19	1.85E-01
SPD_1716		3.28	7.02E-14	4.75	6.97E-29
SPD_1717		3.12	3.92E-12	4.10	1.56E-36
SPD_1718		3.54	5.69E-28	2.66	2.39E-30
SPD_1746		0.98	8.86E-01	2.22	1.82E-09
SPD_1747		0.69	1.70E-02	1.22	4.80E-02
SPD_1748		0.72	1.91E-01	1.42	1.62E-01
SPD_1749		0.92	5.67E-01	1.47	5.86E-03
SPD_1750		1.21	2.86E-01	0.94	7.26E-01
SPD_1751		1.11	6.16E-01	1.15	4.85E-01
SPD_1752		0.93	6.08E-01	1.00	9.95E-01
SPD_1753		0.87	1.39E-01	1.00	9.75E-01
SPD_1754		0.77	5.84E-03	0.86	7.90E-02
SPD_1773	*yidC/oxa*	2.36	6.79E-06	1.45	8.41E-05
SPD_1797	*CcpA/lacI*	0.72	8.53E-03	0.97	7.26E-01
SPD_1798	*desR*	2.05	2.47E-10	2.73	2.41E-21
SPD_1799	*desK*	2.20	8.31E-14	3.06	9.32E-28
SPD_1800		2.60	3.26E-07	4.26	1.58E-26
SPD_1801		3.46	9.18E-19	6.52	2.74E-47
SPD_1802		3.84	8.12E-08	8.19	1.58E-18
SPD_1803		2.93	1.41E-07	6.71	5.22E-14
SPD_1830	*bguA*	5.57	7.89E-27	16.04	4.47E-54
SPD_1831	*bguD*	5.87	2.62E-21	15.94	5.73E-47
SPD_1832	*bguB*	6.87	3.47E-26	17.73	3.59E-48
SPD_1833	*bguC*	5.97	3.40E-20	24.99	3.62E-47
SPD_1952		1.37	3.39E-02	2.04	3.68E-06
SPD_1954		1.64	4.24E-02	2.70	4.17E-04
SPD_1956	*ilvD*	4.75	1.57E-50	9.46	6.78E-255
SPD_1962		1.11	3.38E-01	2.43	8.77E-20
SPD_2018		1.34	5.48E-03	2.11	9.76E-15
SPD_2069		0.77	8.85E-03	2.23	2.79E-19
Down regulated					

SPD_0091		0.25	1.46E-11	0.14	1.71E-80
SPD_0104	*lysM*	0.62	0.0487	0.31	2.93E-19
SPD_0139		0.52	0.00678	0.35	1.09E-10
SPD_0140		0.45	0.000974	0.30	1.33E-13
SPD_0150		0.65	0.0331	0.28	3.33E-31
SPD_0151		0.60	0.00843	0.28	5.57E-33
SPD_0249		0.42	1.86E-16	0.33	5.4E-22
SPD_0303		0.36	8.75E-09	0.34	1.85E-15
SPD_0373		0.50	0.000154	0.24	1.19E-27
SPD_0439		0.52	0.0000298	0.37	1.67E-25
SPD_0466		0.37	3.6E-10	0.27	2.59E-20
SPD_0546	*brnQ*	0.28	7.17E-08	0.36	2.07E-32
SPD_0617		0.47	0.000117	0.35	2.03E-09
SPD_0619		0.53	0.000057	0.26	4.19E-38
SPD_0632	*thiD*	0.45	0.00000605	0.30	2.02E-38
SPD_0885	*ccdA*	0.47	1.74E-11	0.33	1.35E-26
SPD_0886		0.43	3.1E-18	0.29	2.04E-29
SPD_1041	*nrdH*	0.76	0.173	0.34	1.23E-08
SPD_1083		0.54	1.34E-08	0.32	1.61E-40
SPD_1086	*mutY*	0.52	8.89E-22	0.35	5.95E-46
SPD_1263		0.50	0.0000018	0.30	9.27E-29
SPD_1264		0.53	0.0000119	0.35	1.29E-19
SPD_1265		0.52	0.0000421	0.35	7.41E-13
SPD_1327	*bta*	0.39	1.29E-41	0.32	3E-21
SPD_1360		0.37	1.66E-09	0.26	3.49E-14
SPD_1408		0.17	6.2E-67	0.13	2.55E-106
SPD_1415		0.17	6.2E-67	0.25	9.24E-36
SPD_1464	*psaD*	0.41	1.01E-11	0.23	2.43E-27
SPD_1626	*xth*	0.31	3.91E-40	0.36	4.78E-15
SPD_1638		0.77	0.18737787	1.13	0.174
SPD_1649	*piuB*	0.08	1.25E-77	0.08	1.11E-65
SPD_1650	*piuC*	0.12	2.38E-53	0.08	2.76E-94
SPD_1651	*piuD*	0.10	1.16E-70	0.05	1.04E-114
SPD_1652	*piuA*	0.06	2.65E-73	0.04	1.33E-239
SPD_1673	*gtfA*	0.28	3.07E-34	0.32	2.21E-43
SPD_1674		0.32	2.16E-11	0.39	2.02E-10
SPD_1675	*rafG*	0.35	1.56E-14	0.37	4.62E-16
SPD_1676	*rafF*	0.31	3.12E-14	0.46	1.01E-08
SPD_1677	*rafE*	0.29	2.48E-20	0.44	2.21E-15
SPD_1678	*aga*	0.33	1.2E-28	0.33	6E-41
SPD_1774	*pflA*	0.48	4.77E-10	0.33	3.11E-41

SPD_1898		0.68	0.0882	0.26	1.66E-09
SPD_1899		0.81	0.288	0.25	2.03E-24
SPD_2011	*glpF*	0.43	0.00000044	0.36	4.74E-14
SPD_2012	*glpO*	0.50	0.000141	0.33	1.5E-19
SPD_2013	*glpK*	0.48	0.00000925	0.33	1.65E-18
SPD_2014		0.33	1.94E-09	0.18	6.64E-135
SPD_2037	*cysK*	0.42	0.0000188	0.17	2.69E-29
SPD_2038		0.50	0.00491	0.12	6.32E-64

[a]**Fold change:** Differentially expressed genes with fold change ≥ 2 in either one- or two-hour post hemoglobin treatment are shown here.
